# Oral iron supplementation ameliorated alterations in iron uptake and utilization in copper-toxic female Wistar rats

**DOI:** 10.5935/1518-0557.20230014

**Published:** 2023

**Authors:** Bernard Omokheshi Adele, Ogochuckwu F. Eddie-Anunobi, Elsie O. Adewoye

**Affiliations:** 1 Applied and Environmental Physiology Unit, Department of Physiology, Faculty of Basic Medical Sciences, University of Ibadan, Nigeria

**Keywords:** Copper toxicity, Iron supplementation, iron absorption and mobilization, haematology

## Abstract

**Objective:**

Women are more susceptible to both iron deficiency and copper toxicity due to monthly flow and estrogen action, respectively. Oral iron is beneficial for menstruating women and enhances erythropoiesis, but both deficiency and excess of copper impact iron absorption and mobilization. The aim of this study was to investigate the possibility of mitigating copper toxicity in female Wistar rats while supplementing with iron.

**Methods:**

20 female rats (160-180g) were grouped into four: Groups 1 (Control) received 0.3mls normal saline, 2- copper-toxic (100m mg/kg Copper sulphate), 3- Copper-toxic+Iron (100 mg/kg Copper sulphate + 1 mg/kg Ferrous sulphate) and 4- Iron (1 mg/kg Ferrous sulphate). All treatment was administered orally for 5 weeks. Blood was collected retro-orbitally after light anesthesia into EDTA and plain bottles for hematological, serum copper, iron, ferritin and total iron binding capacity (TIBC) analysis. Liver was excised for copper and iron levels while bone marrow was harvested for myeloid/erythroid ratio. The data were analyzed by one-Way ANOVA and statistical significance was considered at *p*<0.05.

**Results:**

Iron supplementation significantly increased packed cell volume, hemoglobin concentration, red blood cell count and myeloid/erythroid ratio, compared to the copper-toxic group. Serum iron and TIBC were significantly increased while liver copper and iron levels reduced significantly in iron supplemented group compared to the copper-toxic group.

**Conclusions:**

Oral iron supplementation mitigated alterations in iron absorption and mobilization following copper toxicity.

## INTRODUCTION

Copper (Cu) is an essential trace element that occurs naturally as organic copper in foodstuff, such as sea food, organ meat (liver), whole grains, vegetables and nuts (Sadhra *et al.*, 2007). Other sources such as electrical wire, cooking utensils and plumbing pipes contain copper in inorganic metallic form. Organic copper present in foodstuff is an essential micro-nutrient vital to the health of all living organisms. It is involved in the metabolism of cholesterol and glucose, formation of red blood cells, absorption and utilization of iron, maintenance of bone, connective tissue and other body organs (Angelova *et al.*, 2011). As a co-factor for a number of enzymes, it helps in the metabolic elimination of free radicals e.g., through Cu-Zn dependent super-oxide dismutase (SOD) (Uauy *et al.*, 1998; Bonham *et al.*, 2002). Excessive copper resulting from elevated levels of copper intake, either from food, hot water pipe, or in inorganic form can lead to toxicity (Ashish *et al.*, 2013). Copper bound to ceruloplasmin mediates bio-availability of ferric iron for heme synthesis and red blood cell formation (Reeves & DeMars, 2004; 2006), making copper deficiency a risk factor of iron-deficiency anemia.

Studies have shown that males and females exhibit significant differences regarding iron status (Kong *et al.*, 2014) which could probably be due to the effect of estrogens and androgens on erythropoiesis (Murphy, 2014). The difference in mean venous hemoglobin levels and red cell mass is generally considered to be caused by a direct stimulatory effect of androgens on erythropoietin production in the kidney, and an inhibitory effect of estrogens on the bone marrow in women (Shahani *et al.*, 2009). Unlike males, the female population is susceptible to low blood iron (Rushton *et al*., 2002; 2009), and the action of estrogen also makes them more susceptible to copper toxicity. Estrogen increases copper retention and its buildup. Exposure to copper toxicity can occur in women from occupational sources, long term intake of foodstuff containing copper, anthropogenic sources, but the use of copper containing contraceptives is also increasing exposure in women. Increased copper level in the body can induce oxidative damage of proteins and lipids (Mattie & Freedman, 2001; Rakshit *et al*., 2018) and challenge erythropoiesis by limiting iron uptake (Ramírez-Cárdenas *et al.*, 2005).

Women are faced monthly with blood and iron loss and developing erythroid cells that need more iron to restore homeostasis (Waldvogel-Abramowski *et al*., 2014) will be affected if they are challenged with copper toxicity. The process of producing new erythrocytes is iron consuming and dependent, hence menstruating women who are known to have low iron level will have the erythropoietic process more impacted during copper toxicity. Fighting iron dyshomeostasis as well as preventing copper toxicity in women with a single therapy that will reduce the burden copper toxicity and iron loss is important. The aim of this study was to investigate the influence of oral iron supplementation on exposure to copper toxicity in female rats.

## MATERIALS AND METHODS

### Experimental design

Twenty female Wistar rats (160-180 g) were used for the study. They were grouped as follows: 1- Control (given 0.3ml of normal saline), 2 - Copper-toxic (Copper sulphate 100mg/kg), 3 - Copper-toxic+Iron (1 mg/kg Ferrous sulphate + Copper sulphate) and 4 - Iron (1 mg/kg ferrous sulphate). Copper sulphate and Ferrous sulphate were administered at an oral dose of 100 and 1 mg/kg respectively for 5 weeks. This study was carried out in the Department of Physiology, University of Ibadan, Nigeria. All protocols followed the guidelines of the University of Ibadan Animal Care and Use in Research Ethics Committee (UI-ACUREC).

### Tissue collection and processing

Blood was collected through cardiac puncture under sodium thiopentone anesthesia into plain and EDTA sample bottles. The EDTA coated blood samples were immediately subjected to hematological assessment using an auto-analyzer (Sysmex Hematology Analyzer, K4500 model). Plain blood samples were allowed for about 30 minutes to clot at room temperature and then centrifuged at 3,500 rpm for 10minutes to separate out serum. Serum was carefully aspirated into new sterile plain bottles for determination of iron (Centronic iron kit, Germany), ferritin, total Iron binding capacity (TIBC), Copper using Fortress diagnostics assay kit, United Kingdom.

After blood collection, liver tissue was harvested, weighed (Camry, model EHA501) and homogenized using a teflon homogenizer in 5 times weight/volume of 10 mM phosphate buffer (pH 7.4). Post-mitochondrial fraction was obtained over centrifugation (10,000 rpm for 15 minutes, 4^o^C) for liver iron (Centronic iron kit, Germany) and copper (Fortress diagnostics assay kit, UK) levels.

Thereafter, the femur was harvested and processed for myeloid-erythroid ratio using H & E staining technique. Bone marrow cell count was first completed and recorded after which the myeloid-erythroid ratio was assessed. This was performed by calculating the total myeloid precursors in proportion to the total erythroid precursors. The myeloid cells alone were counted excluding lymphocytes, monocytes, macrophages, plasma cells, megakaryocytes, osteoblasts, osteoclasts and other myeloid cells.

### Statistical analysis

The values were presented as mean ± standard error of mean (SEM). The data were analyzed using one-way analysis of variance (one-way ANOVA) and statistical significance at *p*<0.05 was established using Newman Keul’s post-hoc test.

## RESULTS

**Figure 1:**
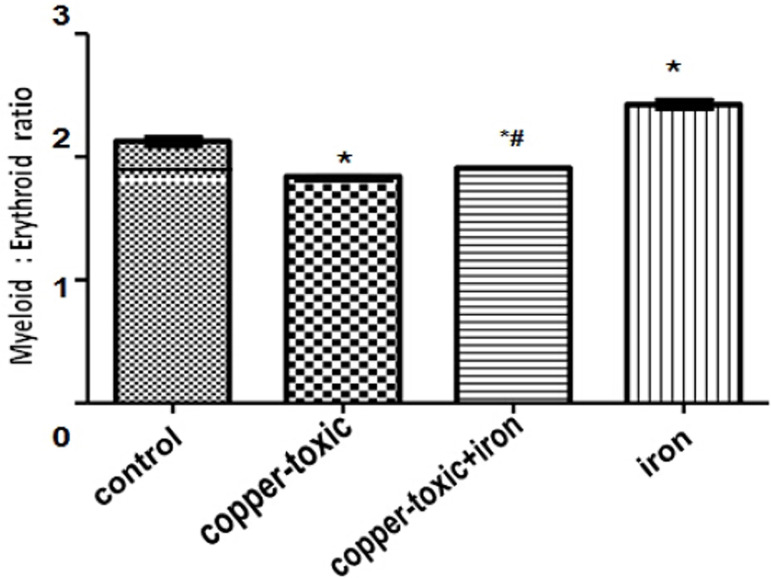
Myeloid/erythroid ratio in iron supplemented rats

Oral iron significantly increased the packed cell volume, hemoglobin concentration, red blood cell count (Table 1) and white blood cell count (Table 2) in group 3 compared to the copper-toxic group. Lymphocyte count (63.00±4.20 *vs*. 60.00±2.90) was increased, while neutrophils (31.00±3.90 *vs*. 37.00±3.60%) reduced significantly in Group 3 compared to Group 2 (Table 2). Myeloid erythroid ratio differs significantly in Group 3 compared to Group 2 (Figure 1).

**Table 1:** Red blood cell indices in iron supplemented rats.

Experimental groups	PCV (%)	Hb (g/dL)	RBC count (x103/mm^3^)
Control	34.00±1.40	11.00±0.44	5.60±0.20
Copper-toxic	28.00±0.66^*^	9.30±0.07^*^	4.50±0.01^*^
Copper-toxic+Iron	40.00±1.60^* #^	13.00±0.51^#^	6.90±0.48^#^
Iron	37.00±1.30	13.00±0.47	6.30±0.26

Values are Mean±SEM; n=5; *p*<0.05

* indicates significance difference compared to control

# indicates significance difference compared to copper-toxic.

**Table 2:** White blood cell indices in iron supplemented rats.

Experimental Groups	WBC (mm^3^)	Lymphocytes (%)	Neutrophils (%)	Monocytes (%)
Control	6310.00±741.00	67.00±1.90	29.00±2.30	2.20±0.49
Copper-toxic	3630.00±589.00^*^	60.00±2.90^*^	37.00±3.60^*^	2.40±0.40
Copper-toxic+Iron	4970.00±639.00	63.00±4.20	31.00±3.90^#^	3.00±0.32
Iron	6520.00±527.00	63.00±4.40	33.00±4.30^#^	2.00±0.55

Values are Mean±SEM; n=5; *p*<0.05

* indicates significance difference compared to control

# indicates significance difference compared to copper-toxic.

Values are Mean±SEM; n=5; *p*<0.05

*indicates significance difference compared to control

^#^indicates significance difference compared to copper-toxic.

Mean body weight increased by 7.24 % while relative liver weight decreased by 5.25% in copper-toxic + iron group compared to copper-toxic alone (Table 3).

**Table 3:** Mean weight change and relative liver weight in iron supplemented rats.

Experimental groups	Body weight (g)	Relative Liver weight
Control	169.40±6.99	3.97±0.11
Copper-toxic	174.00±7.41	4.21±0.32
Copper-toxic+Iron	186.60±4.43	4.00±0.19
Iron	189.20±4.73	3.57±0.19

Values are Mean±SEM; n=5; *p*<0.05.

**Figure 2:**
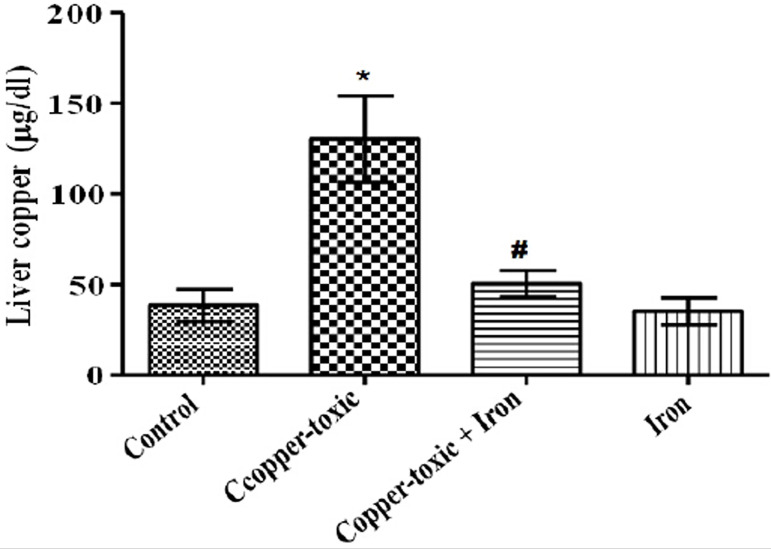
Liver copper level in iron supplemented female rats

**Figure 3:**
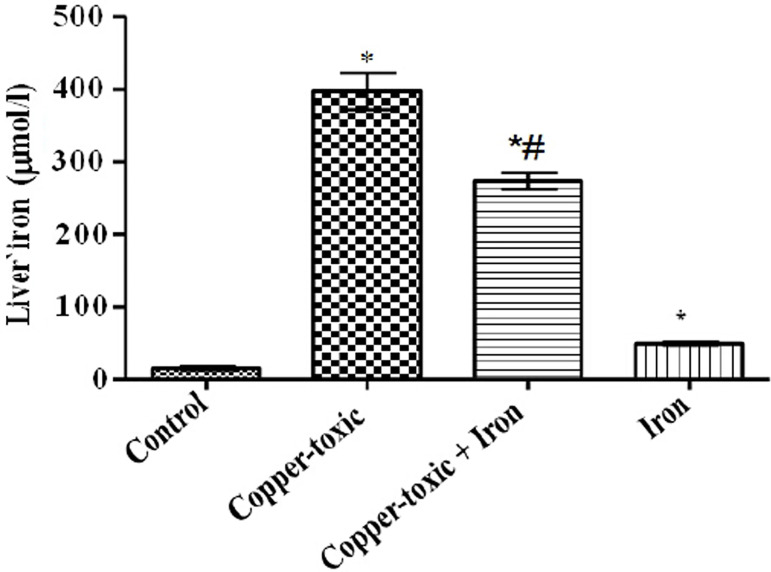
Liver iron level in iron supplemented female rats

**Figure 4:**
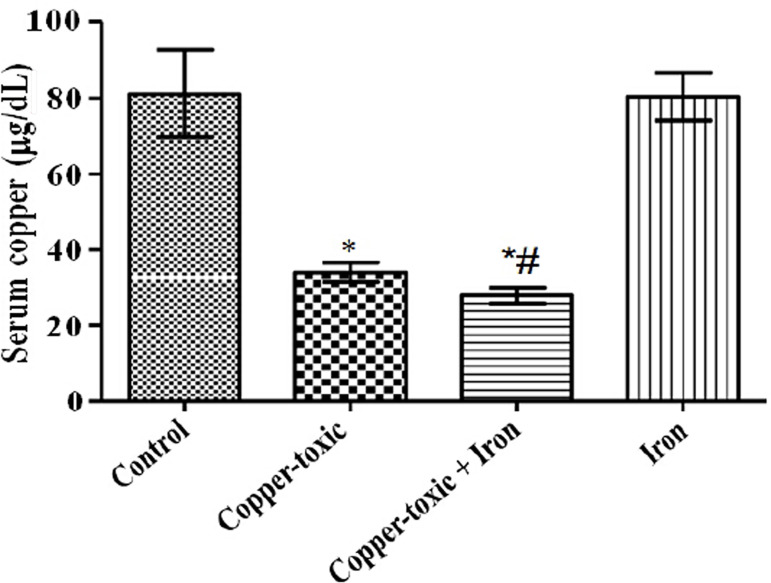
Serum copper level in iron supplemented female rats

**Figure 5:**
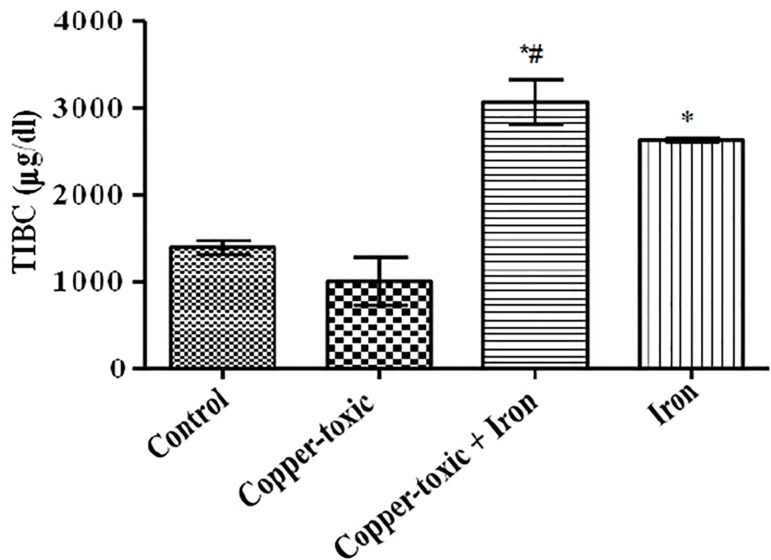
Serum iron level in iron supplemented female rats

Mean Liver copper (Figure 2), iron (Figure 3) and serum copper (Figure 4) levels decreased significantly while serum iron level (Figure 5) increased significantly in Group 3 compared to group 2.

Values are Mean±SEM; n=5; *p*<0.05

*indicates significance difference compared to control

^#^indicates significance difference compared to copper-toxic.

Values are Mean±SEM; n=5; *p*<0.05

*indicates significance difference compared to control

^#^indicates significance difference compared to copper-toxic.

Values are Mean±SEM; n=5; *p*<0.05

*indicates significance difference compared to control

^#^indicates significance difference compared to copper-toxic.

Values are Mean±SEM; n=5; *p*<0.05

*indicates significance difference compared to control

^#^indicates significance difference compared to copper-toxic.

**Figure 6:**
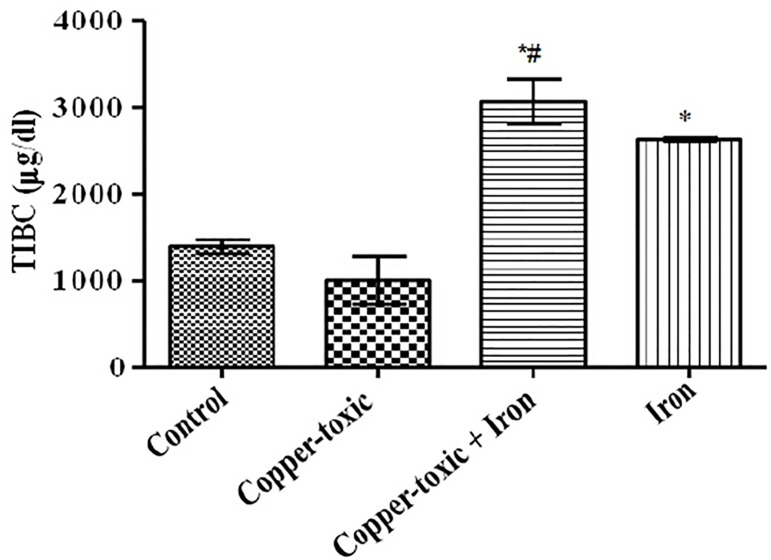
Serum Total Iron Binding Capacity (TIBC) in iron supplemented female rats

**Figure 7:**
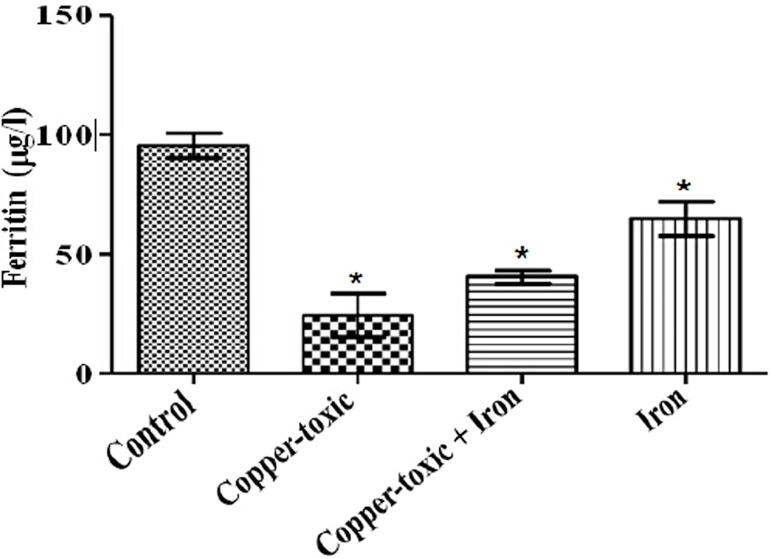
Serum ferritin level in iron supplemented female rats

TIBC (Figure 6) and serum ferritin level (Figure 7) increased following iron supplement in Group 3 compared to Group 2. The increase in TIBC was statistically significant when compared to Groups 1 and 2 (Figure 6).

Values are Mean±SEM; n=5; *p*<0.05

*indicates significance difference compared to control

^#^indicates significance difference compared to copper-toxic.

Values are Mean±SEM; n=5; *p*<0.05

*indicates significance difference compared to control

^#^indicates significance difference compared to copper-toxic.

## DISCUSSION

Copper is physiologically important in many cellular processes such as antioxidant defense, iron absorption and utilization in erythropoietic processes (Bonham *et al.*, 2002; Reeves & DeMars, 2004; 2006). Deviation from the normal copper range either as copper deficiency or copper overload (toxicity) can interrupt some biological processes associated with copper (Ramírez-Cárdenas *et al.,* 2005). Therefore, assessment of the hematological profile will not only provide information on the toxic effect of copper overload but its effect on erythropoietic process. This study showed that copper poisoning significantly caused a decrease in red blood cell indices (count, hematocrit and hemoglobin concentration) and myeloid-erythroid ratio. The decrease could be that copper toxicity leads to reduced iron uptake and utilization, suppression in bone marrow cell differentiation, heme synthesis and increased destruction of red blood cells, which are essential bioactivity of copper *in vivo* (Bonham *et al.*, 2002; Sinkovic *et al.*, 2008). Copper deficiency is known to lead to suppression of the immune system (Bonham *et al.*, 2002). However, findings of this study indicated as decreased WBC and lymphocyte counts following copper toxicity showed that immune system activity could be suppressed and susceptibility to infections increased. Therefore, both copper deficiency and excess could cause immune suppression. Evidence has shown that developing erythroid cells depend on body iron (Waldvogel-Abramowski *et al*., 2014), and development of these cells into mature blood cells will be suppressed by altered iron uptake. Improvement in the hematological profile suggests that oral iron supplementation improved erythropoietic process and hindered uptake of excess copper by bone marrow cells to negatively impact differentiations.

Increase or decrease in organ weights is widely used in the evaluation of toxicities (Wooley, 2003). Alterations in liver weight may suggest treatment related changes including hepatocellular hypertrophy. During copper poisoning, copper is gradually deposited in the liver without producing any significant clinical sign and/or serum copper change (Underwood & Suttle, 1999). In agreement, findings from this study showed an increase in the relative liver weight and copper level in copper toxic rats suggesting that increased deposition of metallic copper ion may have led to the increase in the liver weight. On the contrary, serum copper levels were significantly reduced, which agrees with earlier reports that copper toxicity leads to increased deposition of copper in the liver with no significant increase in the serum (Underwood *& Suttle,* 1999). More so, increase in liver iron indicates alterations in plasma ceruloplasmin level leading to increased iron deposits in the liver (Wood & Han, 1998), thereby increasing liver weight. Oral iron supplementation mitigated bioaccumulation of copper and iron in the liver to reduce liver weight and possibly any damage.

According to Uauy *et al*. (1998), cellular copper levels affect the synthesis of proteins in an organism by enhancing or inhibiting the transcription of specific genes. An abnormality in the transcription of gene coding for iron regulating proteins such as transferrin, ferritin and ceruloplasmin leads to altered iron homeostasis. Disrupted activity of ceruloplasmin alters incorporation of iron into transferrin for use in blood formation (Evans & Abraham, 1973). Low serum iron level and total iron binding capacity (TIBC) following copper toxicity is in agreement with Hedges & Kornegay (1973) and Ramírez-Cárdenas *et al.* (2005) that ingestion of increased copper level results in altered iron absorption, and consequently reduced serum iron levels. Ferritin, in which iron is stored in tissue, reflects iron overload or reduced ceruloplasmin level when serum/plasma level is high (Torti & Torti, 2002), was not affected by excess copper level. Serum iron, ferritin levels and TIBC reflect rates of iron absorption at the duodenum, transferrin activity, iron load and indirectly ceruloplasmin activity (Wood & Han, 1998; Torti & Torti, 2002; Ramírez-Cárdenas *et al.,* 2005). This study also supports earlier work that excess of copper reduces iron absorption and mobilization for cellular use. However, the iron-supplemented group showed increased serum iron level, ferritin, TIBC and reduced serum copper level. Female population of reproductive age may depend on iron supplements to prevent lower than normal blood iron level and enhance erythropoiesis to restore homeostasis (Rushton *et al*., 2002; 2009). The menstruating women showed lower ferritin level, and iron supplementation is advised (Waldvogel-Abramowski *et al*., 2014). Increase in serum iron and ferritin level following oral supplements reflects restoring of homeostasis compared to the control level. Mobilization and transportation of iron and, lowering of excess copper in the serum were possibly enhanced to restore iron homeostasis.

The findings of this study showed that copper toxicity also affects iron metabolism, blood formation, immunity and specifically myeloid/erythroid ratio. However, oral iron supplementation mitigated against iron dyhomeostasis and improve immunity. The results showed that oral iron supplementation could be undertaken as a single therapy to prevent copper toxicity and as well improve iron status in menstruating women.
